# SSTR PET/CT for skull base low-grade meningioma: a critical tool for accurate gross tumor volume delineation in radiotherapy?

**DOI:** 10.1186/s13014-025-02718-4

**Published:** 2025-09-22

**Authors:** Frederik Fuchs, Sebastian N. Marschner, Jan Hofmaier, Maya Rottler, Indra Hadi, Sebastian H. Maier, Tobias Greve, Adrien Holzgreve, Nathalie L. Albert, Raphael Bodensohn, Claus Belka, Maximilian Niyazi, Franziska Walter

**Affiliations:** 1 Department of Radiation Oncology, LMU University Hospital, LMU Munich, Munich, Germany; 2 German Cancer Consortium (DKTK), Partner Site Munich, Munich, Germany; 3 Department of Radiation Oncology, Hospital Lüneburg, Lüneburg, Germany; 4 Department of Neurosurgery, LMU University Hospital, LMU Munich, Munich, Germany; 5 Department of Nuclear Medicine, LMU University Hospital, LMU Munich, Munich, Germany; 6 Ahmanson Translational Theranostics Division, David Geffen School of Medicine, University of California Los Angeles (UCLA), Los Angeles, USA; 7 Department of Radiation Oncology, University Hospital Tübingen, Tübingen, Germany; 8 Bavarian Cancer Research Center (BZKF), Munich, Germany; 9 Center for Neuro-Oncology, Comprehensive Cancer Center Tübingen-Stuttgart, University Hospital Tübingen, Tübingen, Germany; 10 German Cancer Consortium (DKTK), partner site Tübingen, a partnership between DKFZ and University Hospital Tübingen, Tübingen, Germany

**Keywords:** Meningioma, PET/CT, MRI, Radiotherapy, Contouring, Skull base

## Abstract

**Background:**

Precise delineation of gross tumor volume (GTV) is fundamental for effective radiation therapy in low-grade skull base meningiomas. Magnetic resonance imaging (MRI) serves as the primary imaging tool but may not fully represent tumor extent. This study investigates the additional value of incorporating Somatostatin receptor (SSTR)-directed PET/CT in radiation therapy planning.

**Methods:**

A retrospective analysis was conducted with four experienced radiation oncologists contouring GTVs for skull base meningiomas using MRI alone (GTV_MRI), PET/CT alone (GTV_PET/CT), and both modalities combined (GTV_ALL). Consensus ground truth volumes were generated for each modality through a STAPLE algorithm. Agreement between modalities, excluding observer variability, was assessed using statistical metrics including Dice Similarity Coefficient (DSC), Jaccard Index (JCI), Hausdorff distance (HD95), Geographical Miss Index (GMI), sensitivity, and kappa statistics.

**Results:**

The study included 25 patients (15 females, 10 males; median age 56 years (range: 23–74 years), with 96% achieving local control post-radiotherapy over a median follow-up of 64 months (range: 28–135 months). Substantial interobserver agreement was observed, with median kappa values of 0.74 for GTV_MRI, 0.68 for GTV_PET/CT, and 0.77 for GTV_ALL. Median consensus volumes were 6.65 cc (MRI_STAPLE_), 7.21 cc (PET_STAPLE_), and 6.73 cc (ALL_STAPLE_). The median GMI for MRI_STAPLE_ compared to ALL_STAPLE_ was 0.18 (IQR: 0.11–0.39), and 0.21 (IQR: 0.15–0.28) for PET_STAPLE_ compared to ALL_STAPLE_. The DSC indicated the lowest concordance between MRI_STAPLE_ and PET_STAPLE_ with a median of 0.75 (IQR: 0.59–0.82), followed by PET_STAPLE_ versus ALL_STAPLE_ with a median DSC of 0.84 (IQR: 0.79–0.89), and MRI_STAPLE_ versus ALL_STAPLE_ with a median DSC of 0.89 (IQR: 0.76–0.92). The integration of PET/CT with MRI significantly enhanced concordance metrics.

**Conclusion:**

Combining MRI and PET/CT improves GTV delineation in low-grade skull base meningiomas, as PET/CT can reveal regions missed by MRI, which may slightly underestimate tumor size. This multimodal imaging approach enhances consensus and supports its role in radiotherapy planning. Standardized protocols and technical integration remain key future goals.

## Introduction

Skull base meningiomas are frequently treated with postoperative or definitive radiotherapy and present unique challenges for radiotherapy planning due to their diverse locations and close proximity to critical structures, such as the optic chiasm, the optic nerves or the brainstem. Technological advances have led to distinct improvements in radiotherapy planning and delivery. Most notably modern stereotactic treatment options allow for precise dose application with improved sparing of organs at risk. However, this approach requires a high level of accuracy in target volume delineation to avoid marginal tumor misses. Recent contouring guidelines for meningioma aim to standardize radiotherapy delivery and reduce treatment variability [1–3]. Specifically for low grade meningioma the guidelines propose not applying any clinical target volume margin which leaves no room for delineation insecurities. T1-weighted contrast enhanced MRI is the recommended gold standard for gross tumor volume (GTV) delineation due to its excellent soft tissue contrast and detailed anatomical insights. However, its inability to differentiate between tumor tissue and post-treatment changes or edema presents a significant challenge in achieving precise tumor targeting in radiotherapy. PET/CT-imaging with a set of different tracers has evolved as a powerful tool to supplement anatomical imaging, helping to achieve high accuracy in radiotherapy planning. However, metabolic imaging hasn’t been consistently incorporated into delineation guidelines, indicating the need for more thorough investigation.

Research has established the role of metabolic imaging with amino acid tracers in radiotherapy treatment planning for malignant glioma, demonstrating its ability to identify tumor margins [4–6]. The role of PET/CT-imaging in meningioma is a field of interest and several studies have pointed to the importance and effectiveness of these imaging techniques in meningioma management [7–9]. Meningioma have been shown to frequently overexpress somatostatin receptor type 2 (SSTR2) which makes them targetable for SSTR-directed PET/CT and it has been shown to be useful in diagnostics of meningioma [10, 11]. Early studies by Gehler et al., Milker-Zabel et al. and Nyuyki et al. demonstrated [^68^Ga]Ga-DOTA-TOC PET/CT’s effectiveness in enhancing meningioma delineation for radiotherapy planning [12–14]. However, these studies included patients with high-grade and low-grade meningioma. A distinct advantage was found in target definition of osseous infiltration. Furthermore, a large monocenter evaluation revealed that using PET/CT for treatment planning significantly impacted local control in low-grade meningioma [15]. Patients with high-grade meningioma treated with [^68^Ga]Ga-DOTA-TATE PET/CT-based radiotherapy at our institution were evaluated previously by Zollner et al. confirming the value of PET imaging in therapy planning for high-grade meningioma [16].

The current study focuses on radiotherapy planning of low-grade skull base meningioma. We compare MRI-only, SSTR-PET/CT-only, and the combined SSTR-PET/CT + MRI planning approach among 4 physicians and assess the advantages and limitations of the respective modalities as well as the inter-observer variability.

## Methods

### Patients

25 patients with meningioma of the skull base, treated between June 2012 and March 2018 at the Department of Radiation Oncology at LMU University Hospital Munich, were subsequently included. Eight (32%) patients received primary radiotherapy, eleven (44%) patients received postoperative radiotherapy for residual meningioma, and six (24%) patients radiotherapy for local recurrence following prior surgery. In the eleven patients who received postoperative radiotherapy for residual meningioma, a WHO grade 1 meningioma was histologically confirmed according to the WHO classification. Until 2016, the 4th Edition was applied. From 2016 onward, the 4th Revised Edition was used. In cases of radiotherapy for meningioma recurrence, a WHO grade 1 meningioma had been histologically confirmed during the initial surgery at the time of the first diagnosis. For the eight patients who received primary radiotherapy, no histological confirmation was obtained beforehand. In these cases, the diagnosis of meningioma was made based on radiological findings.

The study was approved by the ethical committee of the University Hospital LMU Munich on record number 24–0723.

### Imaging

For all patients, treatment planning involved a planning CT scan with a 1 mm slice thickness in treatment position. Patients were immobilized using a double layered mask (iCAST Head Micro Double 4 mm, IT-V, Innsbruck, Austria). Additionally, contrast-enhanced thin-slice MRI of the skull (T1 +/- contrast, T2) and a SSTR PET/CT scan were performed for treatment planning in all patients. Patients treated from 2012 to 2017 underwent a [^68^Ga]Ga-DOTA-TATE PET/CT scan, while starting from 2018, patients received a [^68^Ga]Ga-DOTA-TOC PET/CT scan for treatment planning. All scans were performed on a Siemens biograph 64 PET/CT (formerly Siemens Medical Solutions, now Siemens Healthineers, Erlangen, Germany). Contrast-enhanced CT scans of the head were performed with 1.5 mL of iopromide (Ultravist^®^-300, formerly Bayer HealthCare, now Bayer Pharmaceuticals, Leverkusen, Germany) per kilogram of body weight. According to clinical guidelines, the injected activity for [^68^Ga]Ga-DOTA-TOC was 100–200 MBq and for [^68^Ga]Ga-DOTA-TATE 150–200 MBq [17]. PET scan of the head was acquired by static emission recording 60–70 min post injection. PET image reconstruction was performed as previously described [18].

### Target definition

The planning CT, MRI sequences, and PET/CT scans were imported into the Elekta Oncentra Treatment Planning (OTP) system (Elekta, Stockholm, Sweden, Version 4.5.2). Within OTP, MRI and PET/CT images were co-registered with the planning CT.

Four experienced radiation oncologists independently created three different GTVs for each of the 25 patients in a sequential process, without knowledge of clinically approved and used target volumes. The first GTV (GTV_MRI) was outlined using only the MRI and planning CT data. The second GTV (GTV_PET/CT) was defined using data from the PET/CT and planning CT, excluding MRI data. The third GTV (GTV_ALL) incorporated data from both MRI and PET/CT scans. For reviewing the PET/CT or MRI scans, no standardized window level or threshold settings was specified. For target volume definition, all visually conspicuous areas were included in the respective GTVs. A threshold-based approach, such as the application of a predefined SUV or an SUV ratio relative to the superior sagittal sinus SUV, was not applied. It was neither possible nor allowed to directly compare the GTVs contoured by the different radiation oncologists at any stage during the contouring process.

### Evaluation of interobserver variability and the impact of MRI, PET/CT on target volume delineation

The agreement among the four radiation oncologists for each modality was assessed using the Kappa statistic, which was calculated using the CERR research treatment planning system [19, 20]. This measure of inter-observer variability can take values from − 1 to + 1, where − 1 indicates no agreement, 0 implies that agreement is no better than chance and + 1 indicates perfect agreement among the observers.

Within each modality, a consensus contour was generated between all four observers by applying the Simultaneous Truth and Performance Level Estimation (STAPLE) algorithm using software tools available in CERR. The STAPLE algorithm computes a probabilistic estimate of the true contour by evaluating each physician’s delineation, as well as an estimated performance level [20, 21]. The consensus contours based on MRI, PET and both modalities were denoted MRI_STAPLE_, PET_STAPLE_ and ALL_STAPLE_, respectively. Individual contours were compared to the STAPLE contour.

The consensus contours for every patient from different modalities were compared using several quantitative geometric measures in the categories volume (absolute), overlap (the Dice Similarity Coefficient (DSC), the Jaccard Conformity Index (JCI), the Geographical Miss Index (GMI) and the Sensitivity) and distance (the 95th percentile Hausdorff Distance (HD95)) [22–27]. All formulas used are summarized in the appendix.

## Results

### Patients and radiation characteristics

15 (60%) female and ten (40%) male patients with a median age of 56 years (range: 23–74 years) were included in our retrospective study. 17 (68%) patients were treated for tumor recurrence or residual tumor after prior surgery, while eight (32%) patients received primary radiotherapy. All tumors were located in the skull base region and were treated with fractionated stereotactic radiotherapy (1.8 Gy to a total dose of 50.4–54.0 Gy). The median follow-up period was 64 months (range: 28–135 months). 96% (24/25) of the patients achieved local control after radiotherapy (Table 1).

**Table 1:** Patient, tumor, and treatment characteristics

Patient	Age (years)	Sex	Localisation	Treatment situation	GTV volume (cc)	IRRT (months)	Dose per fraction (Gy)	Total dose (Gy)
1	48	male	Sphenoid wing with infiltration of the orbit	Residual tumor after resection	21	1	1.8	54.0
2	47	male	Sphenoid wing with infiltraton of cavernous sinus	Residual tumor after resection	5	1	1.8	54.0
3	73	female	Sphenoid wing	Recurrent tumor	1	32	1.8	54.0
4	65	female	Petroclival tentorium with infiltration of the trigeminal cistern	Primary radiotherapy	4	NA	1.8	54.0
5	57	female	Sphenoid wing with infiltration of the orbit	Recurrent tumor	79	29	1.8	54.0
6	44	female	Sphenoid wing	Recurrent tumor	7	114	1.8	54.0
7	69	male	Clinoidal region with invasion of the optic canal	Residual tumor after resection	2	1	1.8	52.2
8	58	male	Temporal along the course of the trigeminal nerve	Primary radiotherapy	7	NA	1.8	54.0
9	74	male	Sphenoid wing with infiltraton of cavernous sinus	Primary radiotherapy	12	NA	1.8	52.2
10	56	female	Foramen magnum	Residual tumor after resection	3	15	1.8	50.4
11	65	female	Sphenoid wing with infiltration of the orbit	Recurrent tumor	48	108	1.8	52.2
12	56	male	Sphenoid wing with infiltration of the cavernous sinus and orbit	Primary radiotherapy	39	NA	1.8	54.0
13	58	female	Sphenoid wing	Primary radiotherapy	1	NA	1.8	54.0
14	57	female	Sphenoid wing	Residual tumor after resection	14	1	1.8	54.0
15	34	female	Petroclival region	Residual tumor after resection	4	6	1.8	54.0
16	29	male	Clinoidal region	Residual tumor after resection	1	2	1.8	52.2
17	47	female	Sphenoid wing with infiltration of the cavernous sinus and orbit	Recurrent tumor	50	89	1.8	54.0
18	56	female	Suprasellar	Primary radiotherapy	3	NA	1.8	52.2
19	59	female	Sinus cavernosus	Residual tumor after resection	11	3	1.8	54.0
20	46	female	Temporopolar region with infiltration of the orbit	Residual tumor after resection	7	1	1.8	52.2
21	68	female	Sphenoid wing with infiltration of the cavernous sinus and orbit	Primary radiotherapy	22	NA	1.8	54.0
22	40	female	Sphenoid wing	Residual tumor after resection	12	2	1.8	52.2
23	45	male	Sphenoid wing with infiltration of the orbit	Residual tumor after resection	29	1	1.8	54.0
24	23	male	Sphenoid wing with infiltration of the cavernous sinus	Recurrent tumor	9	14	1.8	54.0
25	52	male	Temporomesial region with infiltration of the cavernous sinus	Primary radiotherapy	4	NA	1.8	54.0

Abbreviations: IRRT: Interval between tumor resection and start of radiotherapy; NA: Not applicable

### Comparative analysis

**Fig. 1:**
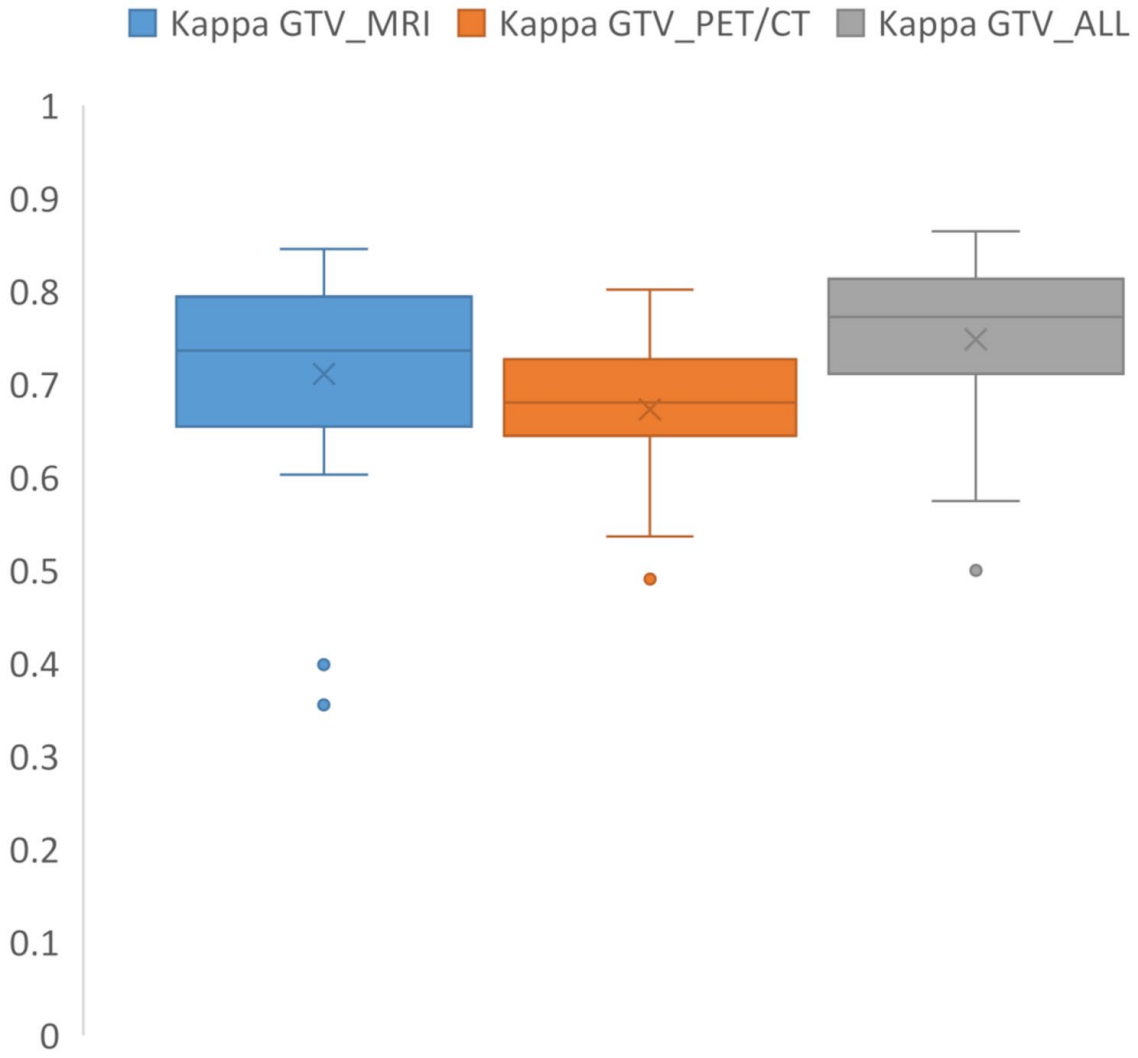
This boxplot displays the Kappa statistic values for the three Gross Tumor Volumes (GTVs) across all 25 patients, as outlined by the four radiation oncologists. The data is segmented based on the imaging technique used: MRI alone (Kappa GTV_MRI), PET/CT alone (Kappa GTV_PET/CT), and a combination of MRI and PET/CT (Kappa GTV_ALL)

**Fig. 2:**
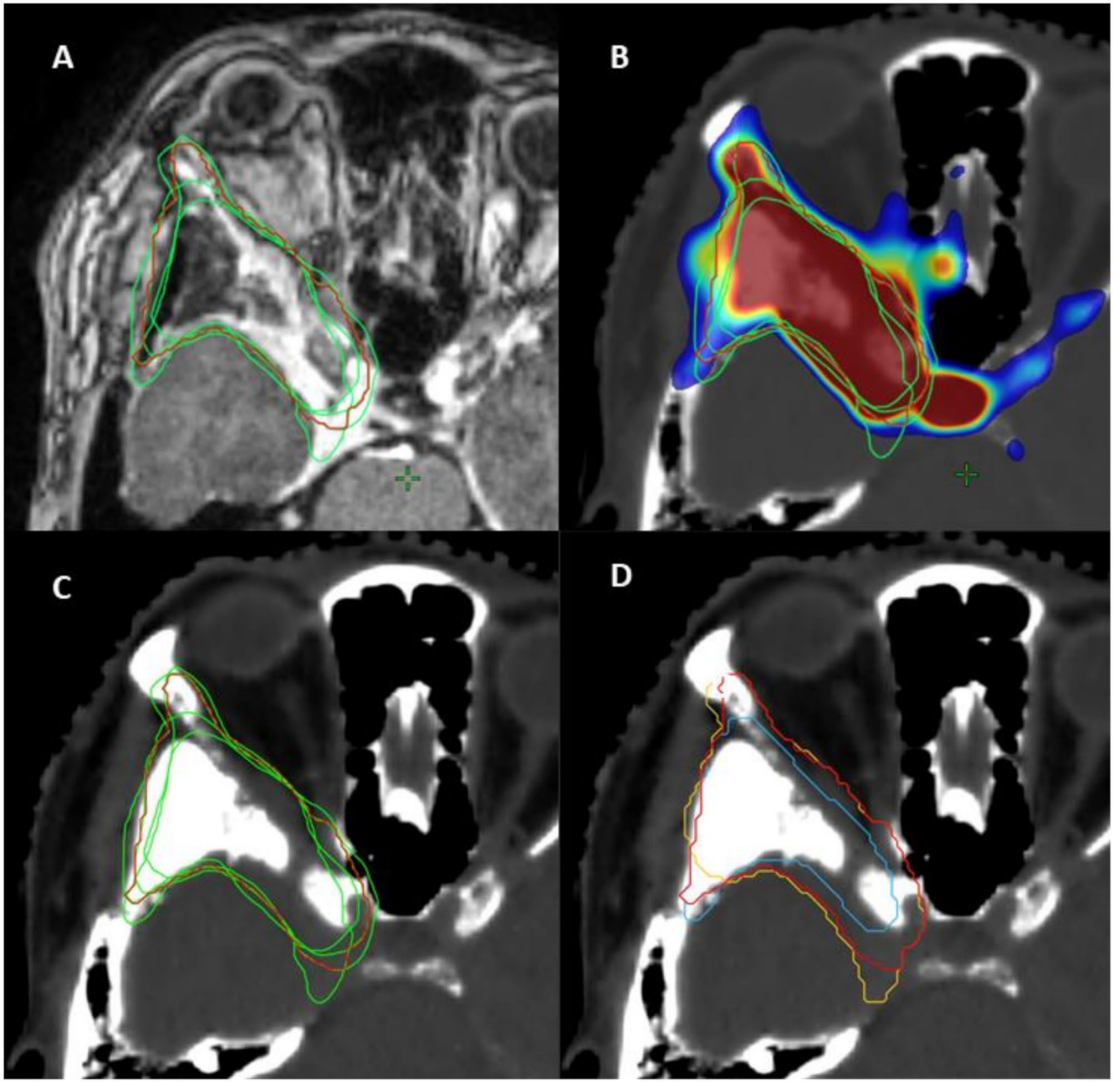
The individual GTV_ALL contours from the four radiation oncologists are shown in green, while the consensus structure (ALL_STAPLE_) is displayed in red: (**A**) on the MRI (T1-weighted with contrast), (**B**) on the SSTR PET, and (**C**) on the planning CT. (**D**) The MRI_STAPLE_ contour is depicted in blue, the PET_STAPLE_ contour in yellow, and the ALL_STAPLE_ contour in red

The agreement among radiation oncologists was tested using Kappa-statistics with a median κ = 0.74 (IQR: 0.66–0.78) for GTV_MRI, κ = 0.68 (IQR: 0.65–0.72) for GTV_PET/CT, and κ = 0.77 (IQR: 0.71–0.81) for GTV_ALL (Fig. 1). An example of the four interrater contours along with the STAPLE contour is shown in Fig. 2A.

**Fig. 3:**
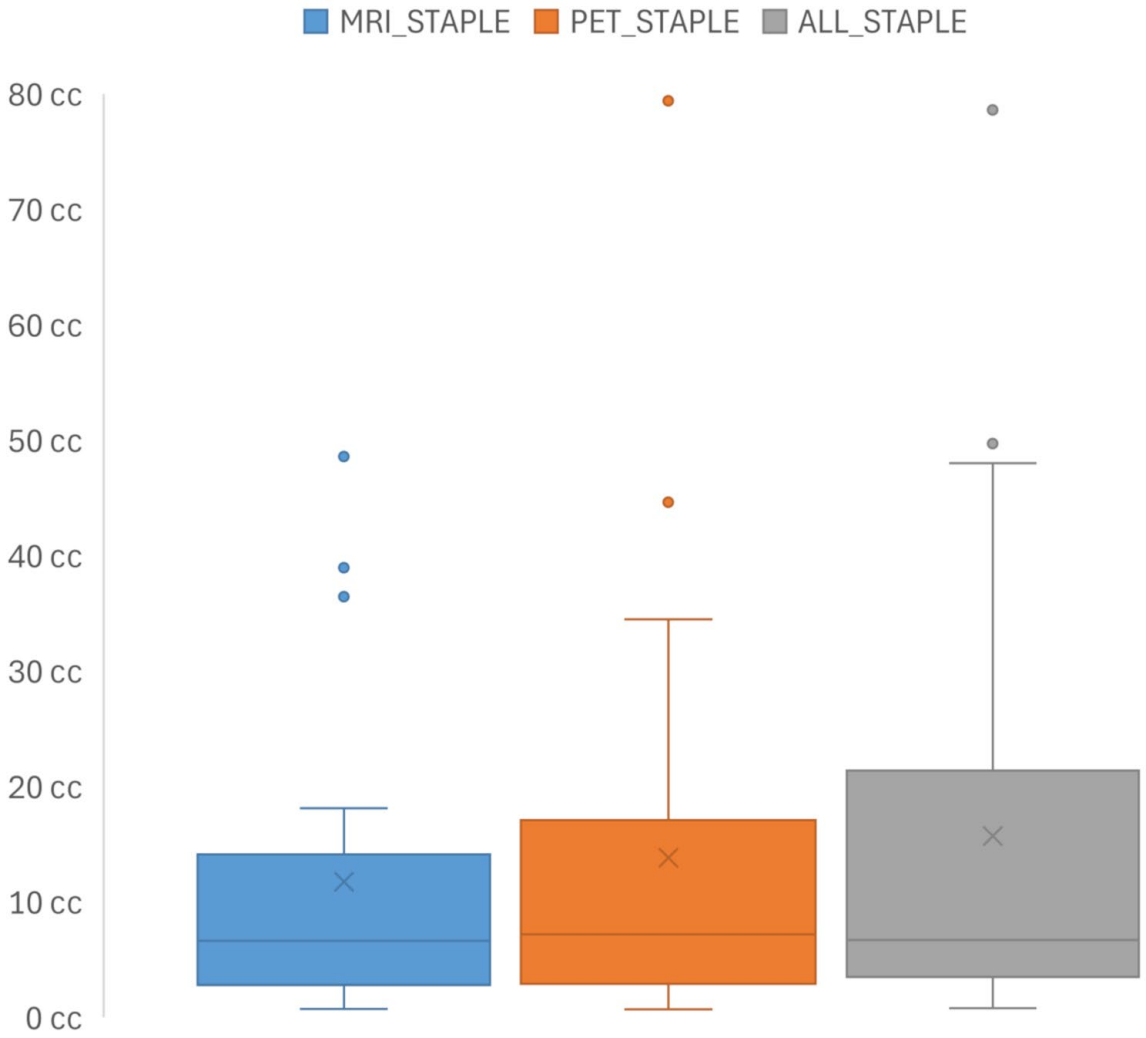
In this boxplot, the volumes of the staple volumes for the GTVs contoured by the four radiation oncologists based on MRI (MRI_STAPLE_), PET/CT (PET_STAPLE_), and MRI and PET/CT combined (ALL_STAPLE_) are depicted

**Fig. 4:**
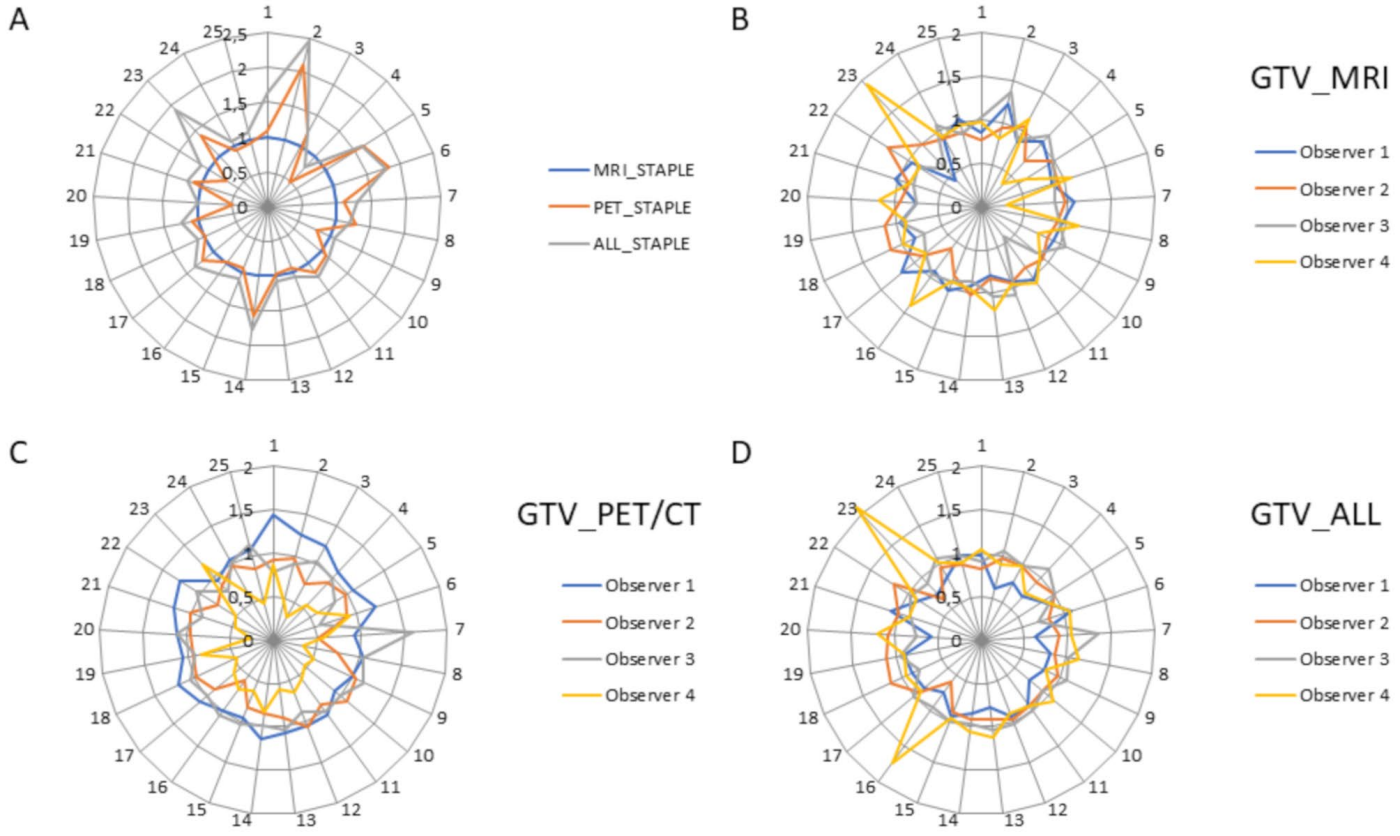
(**A**) volume of the PET_STAPLE_ (orange) and the ALL_STAPLE_ (grey) relative to the MRI_STAPLE_ (blue) volume across all 25 patients. (**B**) volumes of GTV_MRI, (**C**) GTV_PET/CT and (**D**) GTV_ALL for the four individual observers relative to their respective STAPLE volumes (Observer 1 in blue, Observer 2 in orange, Observer 3 in grey, Observer 4 in yellow)

The median volume of the consensus structures from MRI (MRI_STAPLE_) is 6.65 cc (IQR: 3.03–12.91 cc), for PET/CT (PET_STAPLE_) 7.21 cc (IQR: 2.97–13.93 cc), and for the GTV based on MRI and PET/CT (ALL_STAPLE_) 6.73 cc (IQR: 3.72–20.96 cc) (Fig. 3). The staple volumes in relation to MRI_STAPLE_ for each patient are illustrated in Fig. 4A.

The volumes of the individual observers for GTV_MRI, GTV_PET/CT, and GTV_All in relation to the staple volumes MRI_STAPLE_, PET_STAPLE_, and ALL_STAPLE_ for all 25 patients are depicted in Fig. 4B-D.

Lowest concordance among individual GTVs was observed between MRI_STAPLE_ and PET_STAPLE_, indicated by a median Dice Similarity Coefficient (DSC) of 0.75 (IQR: 0.59–0.82). In comparison, MRI_STAPLE_ versus ALL_STAPLE_ showed a median DSC of 0.89 (IQR: 0.76–0.92), respectively, and PET_STAPLE_ versus ALL_STAPLE_ presented a median DSC of 0.84 (IQR: 0.79–0.89). The median Jaccard Coefficient Index (JCI) typically registered lower than the DSC values, with 0.59 (IQR: 0.42–0.69) for MRI_STAPLE_ versus PET_STAPLE_, 0.80 (IQR: 0.61–0.85) for MRI_STAPLE_ versus ALL_STAPLE_, and 0.73 (IQR: 0.66–0.80) for PET_STAPLE_ versus ALL_STAPLE_.

Aligning with the DSC and JCI findings, the greatest discrepancy between the different volumes, as indicated by the HD95, was observed between MRI_STAPLE_ and PET_STAPLE_, with a median distance of 0.66 cm (IQR: 0.35–1.07 cm), respectively. The analysis of MRI_STAPLE_ versus ALL_STAPLE_ recorded a median HD95 of 0.37 cm (IQR: 0.18–0.61 cm), and PET_STAPLE_ versus ALL_STAPLE_ yielded a median HD95 of 0.44 cm (IQR: 0.26–0.72 cm).

GMI and Sensitivity were only reported for comparisons involving ALL_STAPLE_. The GMI showed little variation between MRI_STAPLE_ vs. ALL_STAPLE_ and PET_STAPLE_ vs. ALL_STAPLE_, with medians of 0.18 (IQR: 0.11–0.39) and 0.21 (IQR: 0.15–0.28), respectively. Sensitivity measurements were closely aligned with 0.82 (IQR: 0.61–0.89) for MRI_STAPLE_ vs. ALL_STAPLE_ and 0.79 (IQR: 0.72–0.85) for PET_STAPLE_ vs. ALL_STAPLE_. Detailed calculations of these metrics for each of the 25 patients are provided in Table 1. Figure 2B illustrates the MRI_STAPLE_, PET_STAPLE_, and ALL_STAPLE_ contours on a representative slice from a single patient (Table 2).

**Table 2:** This table displays mean values with standard deviation and median values of comparisons of Dice Similarity Coefficient (DSC), Jaccard-Conformity-Index (JCI), Hausdorff Distance 95th percentile (HD95), Geographical Miss Index (GMI) and Sensitivity across the different STAPLE contours (MRISTAPLE; PETSTAPLE and ALLSTPALE) for all 25 patients

Patient	Dice Similarity Coefficient			Hausdorff Distance (95th percentile)			Jaccard Conformity Index			General Miss Index		Sensitivity	
	MRI _STAPLE_ & PET_STAPLE_	MRI _STAPLE_ & ALL_STAPLE_	PET_STAPLE_ & ALL_STAPLE_	MRI _STAPLE_ & PET_STAPLE_	MRI _STAPLE_ & ALL_STAPLE_	PET_STAPLE_ & ALL_STAPLE_	MRI _STAPLE_ & PET_STAPLE_	MRI _STAPLE_ & ALL_STAPLE_	PET_STAPLE_ & ALL_STAPLE_	MRI _STAPLE_ & ALL_STAPLE_	PET_STAPLE_ & ALL_STAPLE_	MRI _STAPLE_ & ALL_STAPLE_	PET_STAPLE_ & ALL_STAPLE_
**1**	0.53	0.76	0.79	0.78	0.69	0.78	0.36	0.61	0.66	0.39	0.34	0.61	0.66
**2**	0.43	0.57	0.82	1.07	1.00	0.51	0.27	0.40	0.69	0.60	0.24	0.40	0.76
**3**	0.84	0.90	0.88	0.21	0.15	0.18	0.72	0.82	0.78	0.18	0.12	0.82	0.88
**4**	0.55	0.83	0.70	1.22	0.91	0.90	0.38	0.71	0.53	0.05	0.44	0.95	0.56
**5**	0.60	0.72	0.87	1.26	1.03	0.60	0.43	0.56	0.77	0.42	0.13	0.58	0.87
**6**	0.65	0.71	0.92	0.64	0.58	0.20	0.48	0.55	0.86	0.45	0.06	0.55	0.94
**7**	0.48	0.62	0.76	0.82	0.44	0.38	0.32	0.45	0.61	0.45	0.30	0.55	0.70
**8**	0.80	0.90	0.88	0.31	0.20	0.24	0.67	0.82	0.79	0.18	0.09	0.82	0.91
**9**	0.75	0.94	0.80	0.77	0.18	0.73	0.60	0.89	0.66	0.08	0.31	0.92	0.69
**10**	0.72	0.88	0.81	0.35	0.23	0.29	0.56	0.79	0.68	0.18	0.21	0.82	0.79
**11**	0.84	0.89	0.93	0.44	0.39	0.26	0.73	0.80	0.87	0.19	0.10	0.81	0.90
**12**	0.87	0.95	0.90	0.68	0.30	0.62	0.77	0.91	0.81	0.08	0.16	0.92	0.84
**13**	0.75	0.94	0.79	0.21	0.11	0.23	0.59	0.89	0.66	0.09	0.25	0.91	0.75
**14**	0.59	0.70	0.89	1.58	0.97	1.40	0.42	0.54	0.80	0.45	0.16	0.55	0.84
**15**	0.82	0.91	0.84	0.35	0.15	0.26	0.69	0.84	0.73	0.14	0.22	0.86	0.78
**16**	0.70	0.89	0.78	0.32	0.15	0.32	0.54	0.80	0.64	0.18	0.28	0.82	0.72
**17**	0.75	0.84	0.89	0.66	0.61	0.49	0.59	0.73	0.80	0.27	0.15	0.73	0.85
**18**	0.80	0.92	0.82	0.29	0.20	0.25	0.67	0.86	0.70	0.12	0.22	0.88	0.78
**19**	0.77	0.86	0.90	0.53	0.37	0.44	0.63	0.75	0.82	0.23	0.15	0.77	0.85
**20**	0.50	0.94	0.56	1.18	0.15	1.16	0.33	0.89	0.39	0.05	0.58	0.95	0.42
**21**	0.85	0.88	0.95	0.42	0.38	0.21	0.74	0.79	0.90	0.19	0.09	0.81	0.91
**22**	0.67	0.92	0.76	0.80	0.47	0.72	0.51	0.85	0.61	0.13	0.38	0.87	0.62
**23**	0.50	0.54	0.83	1.08	0.69	0.51	0.34	0.37	0.70	0.59	0.28	0.41	0.72
**24**	0.82	0.92	0.87	1.75	0.24	1.55	0.70	0.85	0.77	0.11	0.20	0.89	0.80
**25**	0.82	0.94	0.85	0.38	0.15	0.32	0.70	0.88	0.74	0.11	0.21	0.89	0.79
**Median**	0.75	0.89	0.84	0.66	0.37	0.44	0.59	0.80	0.73	0.18	0.21	0.82	0.79
**Min**	0.43	0.54	0.56	0.21	0.11	0.18	0.27	0.37	0.39	0.05	0.06	0.40	0.42
**25th Percentile**	0.59	0.76	0.79	0.35	0.18	0.26	0.42	0.61	0.66	0.11	0.15	0.61	0.72
**75th Percentile**	0.82	0.92	0.89	1.07	0.61	0.72	0.69	0.85	0.80	0.39	0.28	0.89	0.85
**Max**	0.87	0.95	0.95	1.75	1.03	1.55	0.77	0.91	0.90	0.60	0.58	0.95	0.94

## Discussion

The aim of the current study was to evaluate the value of SSTR PET for treatment planning of low grade skull base meningioma. By introducing STAPLE contours as ground truth volumes — created as consensus structures using a STAPLE algorithm for each imaging modality — we were able to apply statistical measures of agreement between imaging modalities rather than focusing on individual observer variability, and performed further comparative statistics using the STAPLE contours to compare the performance of the different imaging modalities.

Our findings indicate a high concordance of PET, as evidenced by an average Dice Similarity Coefficient (DSC) of 0.83 and the lowest observed standard deviation of 0.08 in comparison with ALL_STAPLE_. Furthermore, the elevated Jaccard Index (JCI) scores in the comparison between PET_STAPLE_ and ALL_STAPLE_ further underscore the significance of PET in accurately delineating meningiomas.

These results align with previous research by Perlow et al. and Kowalski et al. which demonstrated that [^68^Ga]Ga-DOTA-TATE PET imaging enhances treatment planning by identifying regions that MRI may miss, such as intraosseous involvement, falcine extensions, or satellite lesions [28, 29]. Moreover, PET/CT, particularly with tracers like [^68^Ga]Ga-DOTA-TOC and [^68^Ga]Ga-DOTA-TATE, complements MRI by providing metabolic insights into tumor tissues, as noted by Milker-Zabel et al. [13]. Research by Maclean et al. and Pelak et al. further demonstrated PET/CT’s effectiveness in reducing interobserver variability in tumor contouring, thereby improving treatment uniformity and facilitating the development of standardized protocols especially inside the skull base [30, 31]. Maclean et al. additionally reported comparable Kouwenhoven conformity levels for PET/MRI and PET/CT when each was combined with CT and MRI, indicating no further advantage for PET/MRI.

While the high JCI scores between PET_STAPLE_ and ALL_STAPLE_ favor the PET/CT contour, the lower values observed when comparing PET/CT-only contours with MRI-only contours suggest that each modality delineates different aspects of the meningioma. Notably, the superior median JCI values associated with MRI_STAPLE_ indicate the role of MRI in contouring skull base meningiomas.

Additionally, MRI contours in our analysis exhibited the most homogeneous results compared to ALL_STAPLE_, with a mean DSC of 0.84 and a JCI of 0.73 among different observers. The HD95 metric displayed the smallest mean distances at only 0.43 cm, with a standard deviation of 0.3 cm. This indicates that MRI_STAPLE_ contours achieved the most uniform outcomes with the highest median values. These findings are consistent with the study by Morimoto et al., where 18 radiation oncologists contoured GTVs for 13 cases of canine intracranial meningioma [32]. Their research reported improved interobserver agreement metrics, such as DSC and Conformity Index, when using MRI.

However, we observed that MRI tends to slightly underestimate the actual size of meningiomas, as illustrated in Figs. 3 and 4a. This underestimation is further supported by GMI and sensitivity metrics, with a median GMI of 0.18 (IQR: 0.11–0.39) for MRI_STAPLE_ compared to ALL_STAPLE_, indicating a consistent yet modest underestimation relative to the consensus volume. This limitation would be particularly evident in post-treatment settings, where distinguishing between recurrent disease and treatment-related changes is critical. In these scenarios, PET/CT has been shown to improve the differentiation of tumor tissues from surrounding structures, effectively addressing MRI’s shortcomings, as demonstrated by Gehler et al. and Nyuyki et al. [12, 14].

Comparison of the STAPLE volumes demonstrates that the ALL_STAPLE_ contour lies between the smaller MRI_STAPLE_ and the larger PET_STAPLE_ contour, indicating that ALL_STAPLE_ represents a balanced integration of MRI and PET data rather than a simple summation. Given the complexity of GTV delineation for meningiomas, our sample size of 25 cases might be insufficient to definitively determine whether PET information leads to an increase or decrease in target volume. This effect may depend on factors such as whether patients are newly diagnosed or have recurrent disease with possible scar tissue or post-therapeutic changes, as well as tumor localization. This variability might explain why previous studies have reported differing results regarding changes in target volume with PET imaging. For instance, a decrease in target size was reported by Graef et al. in 8 patients with optic nerve sheath meningiomas undergoing PET/MRI prior to radiosurgery [33]. Similarly, Mahase et al. reported a statistically significant decrease in planning target volumes for postoperative radiotherapy in 8 out of 29 patients undergoing PET/MRI [34]. Conversely, Gehler et al. reported larger GTV volumes based on PET in 50% of cases compared to MRI in a study of 26 patients, while Graef et al. found that the addition of [^68^Ga]Ga-DOTA-TOC PET resulted in more than a 10% modification of GTV size in 67% of the 48 included meningiomas [12, 33]. To fully assess this question, a study with a larger sample size and subgroup analyses according to exact tumor localization and other factors like osseous infiltration and proximity to organs at risk would be needed.

Despite the advantages of PET/CT, we also found variability in PET-based contour delineation, reflected by an HD95 of 0.54 ± 0.37 cm, indicating greater variability compared to MRI-based contours. Kappa statistics further revealed a kappa value of 0.67 ± 0.08 for GTV_PET/CT, slightly lower than the 0.71 ± 0.12 for GTV_MRI. This suggests slightly higher variability among oncologists in delineating GTV_PET/CT, although both modalities demonstrated substantial agreement. The variability in GTV_PET/CT is likely influenced by the subjective nature of PET signal windowing, compounded by the absence of standardized windowing protocols, absolute SUV thresholds, or established relative threshold values (e.g., relative to the sinus sagittalis superior). This subjectivity, particularly around regions like dural tails, contributes to the observed discrepancies.

Consequently, integrating MRI and PET/CT significantly enhances the accuracy of tumor boundary definition in meningiomas, as demonstrated by Acker et al. [33, 35].

Our findings support this integration, showing that the overlap between MRI_STAPLE_ and PET_STAPLE_ accounted for a mean of 58.65% of the ALL_STAPLE_ contour. Further analysis indicated that the remaining ALL_STAPLE_ volume comprised approximately 18% MRI-specific and 19% PET-specific volumes. This underscores that each modality captures distinct tumor regions, emphasizing the importance of both their individual and combined contributions in comprehensive tumor delineation.

Notably, when MRI and PET/CT were combined to create the ALL_STAPLE_ contour, the highest kappa value was achieved (mean 0.75 ± 0.09). This finding underscores that integrating all available imaging information leads to stronger consensus among clinicians regarding tumor boundaries. It suggests that a multimodality approach promotes more accurate and consistent tumor delineation, which is essential for effective radiation therapy planning. The combined use of [^68^Ga]Ga-DOTA-TOC PET and MRI significantly improves target definition accuracy, as reported by Guinto-Nishimura et al. and d’Amico et al. [36, 37]. Similarly, Maclean et al. and Pelak et al. emphasized the role of simultaneous PET/MRI in reducing interobserver variability in tumor contouring, which is critical for consistent and effective radiotherapy [30, 31].

These kappa value analyses highlight the importance of developing robust and standardized protocols for GTV delineation in radiation oncology. Utilizing composite assessments from multiple imaging modalities could achieve higher concordance rates in clinical practice, ultimately improving patient outcomes.

While the integration of MRI and PET/CT improves tumor delineation accuracy, challenges remain. Technical difficulties with image fusion, coordinating scans, and increased time and resource demands have been noted by Thorwarth et al. and Stade et al. [38, 39]. Additionally, high costs and limited availability of advanced PET tracers can limit broader adoption, especially in resource-constrained settings. Despite these limitations, the field is progressing, with new tracers like [^68^Ga]-FAPI04 showing promise in refining the role of PET/CT in meningioma management, as highlighted by Bi et al. and Denizmen et al. [40, 41].

A key strength of our study is the involvement of multiple experienced radiation oncologists in the tumor contouring process. This approach enhances the reliability of our results by reducing bias and improving contouring consistency, lending further confidence to our findings. Although we lacked a true gold-standard contour for comparison and instead used a consensus contour derived from individual inputs — which could introduce variability depending on the quality of the individual contours — the high conformity among these contours supports the robustness of our results. Therefore, we do not consider this a significant limitation, especially given the expertise of the physicians involved.

However, our study has several other limitations. Its retrospective nature and the small patient cohort may limit the generalizability of our findings. Nevertheless, we believe our results offer a valuable contribution to the growing body of literature on the use of PET imaging in meningioma treatment planning, for several reasons. First, we specifically focused on low-grade meningiomas, whereas most previous studies included mixed-grade populations, often emphasizing high-grade cases. Second, to our knowledge, this is the first study to apply the STAPLE algorithm to evaluate target volume agreement in this context. While prior research has highlighted the role of PET in detecting osseous infiltration in high-grade meningiomas, our findings suggest that metabolic imaging also provides significant benefits for GTV delineation in low-grade meningiomas. Despite the limited sample size, the use of a standardized, reproducible analysis framework adds methodological strength to our findings and our results provide valuable insights into the role of PET/CT in treatment planning for low-grade meningiomas.

## Conclusion

Our study underscores the role of SSTR PET in serving as a complementary tool that improves contouring consistency and reduce inter-observer variability in low-grade skull base meningiomas. While MRI remains essential for consistent contouring with high interobserver agreement, SSTR PET adds valuable information that can identify regions potentially overlooked by MRI alone. This emphasizes the importance of a multimodality approach in radiation therapy planning. Future efforts should focus on developing standardized protocols for PET imaging and addressing technical and logistical challenges to optimize its integration into clinical practice.

## Data Availability

No datasets were generated or analysed during the current study.

## Appendix

The metrics used in this work were calculated as follows:

- *Dice Similarity Coefficient (DSC)* = $\:\frac{2(A\cap\:B)}{A+B}$ [22].
- *Jaccard Conformity Index (JCI)* = $\:\frac{A\cap\:B}{A\cup\:B}$ [23, 24].
- *Geographical Miss Index (GMI)* = $\:\frac{B-(A\cap\:B)}{B}$ [27].
- *Sensitivity* = $$\:\frac{A\cap\:B}{B}$$ [26].
  *A represents the MRI*_*STAPLE*_ *or PET*_*STAPLE*_ *volume*.
  *B represents the ALL*_*STAPLE*_ *volume*.
  *A*∩*B represents the intersection of A and B*.
  *A*∪*B represents the union of A and B*.
- *95th percentile Hausdorff Distance (HD95)* = *95th percentile* [*d*(*S*, *S*´),*d*(*S´*,*S*)] [25].
- *95th percentile of surface distances between segmentation S and S*′, *where d(S*, *S*′*) and d(S*′, *S) denote the shortest distances between corresponding surface points.*
